# The influence of Arctic Fe and Atlantic fixed N on summertime primary production in Fram Strait, North Greenland Sea

**DOI:** 10.1038/s41598-020-72100-9

**Published:** 2020-09-17

**Authors:** Stephan Krisch, Thomas J. Browning, Martin Graeve, Kai-Uwe Ludwichowski, Pablo Lodeiro, Mark J. Hopwood, Stéphane Roig, Jaw-Chuen Yong, Torsten Kanzow, Eric P. Achterberg

**Affiliations:** 1 Marine Biogeochemistry Division, GEOMAR Helmholtz Centre for Ocean Research, 24148 Kiel, Germany; 2 Alfred-Wegener-Institute for Polar and Marine Research, 27570 Bremerhaven, Germany; 3 Faculty of Science and Marine Environment, Universiti Malaysia Terengganu, 21030 Kuala Nerus, Terengganu, Malaysia

**Keywords:** Marine chemistry, Element cycles

## Abstract

Climate change has led to a ~ 40% reduction in summer Arctic sea-ice cover extent since the 1970s. Resultant increases in light availability may enhance phytoplankton production. Direct evidence for factors currently constraining summertime phytoplankton growth in the Arctic region is however lacking. GEOTRACES cruise GN05 conducted a Fram Strait transect from Svalbard to the NE Greenland Shelf in summer 2016, sampling for bioessential trace metals (Fe, Co, Zn, Mn) and macronutrients (N, Si, P) at ~ 79°N. Five bioassay experiments were conducted to establish phytoplankton responses to additions of Fe, N, Fe + N and volcanic dust. Ambient nutrient concentrations suggested N and Fe were deficient in surface seawater relative to typical phytoplankton requirements. A west-to-east trend in the relative deficiency of N and Fe was apparent, with N becoming more deficient towards Greenland and Fe more deficient towards Svalbard. This aligned with phytoplankton responses in bioassay experiments, which showed greatest chlorophyll-a increases in + N treatment near Greenland and + N + Fe near Svalbard. Collectively these results suggest primary N limitation of phytoplankton growth throughout the study region, with conditions potentially approaching secondary Fe limitation in the eastern Fram Strait. We suggest that the supply of Atlantic-derived N and Arctic-derived Fe exerts a strong control on summertime nutrient stoichiometry and resultant limitation patterns across the Fram Strait region.

## Introduction

The contribution of the Arctic Ocean to global primary production is ~ 1%^1,2^ but has increased by ~ 30% between 1998–2012 as a result of surface ocean warming and an associated increase in ice-free ocean surface area^3^. Iron (Fe) and fixed nitrogen (N) are essential nutrients for phytoplankton, co-regulating growth alongside light availability throughout much of the global ocean^4–8^. Whilst parts of the high latitude North Atlantic (> 50°N) feature Fe limited phytoplankton growth in spring-summer^9,10^, summertime Arctic primary production is widely thought to be limited by fixed N availability^11–14^ due to low fixed N concentrations across the Arctic basin^15^. Direct experimental assessments of (micro)nutrients limiting to phytoplankton growth in the Arctic Ocean are however sparse, despite the pronounced impacts of climate change on this region^16–18^, potentially altering nutrient fluxes and promoting Fe limitation in the Arctic Ocean^11,19^.

**Figure 1:**
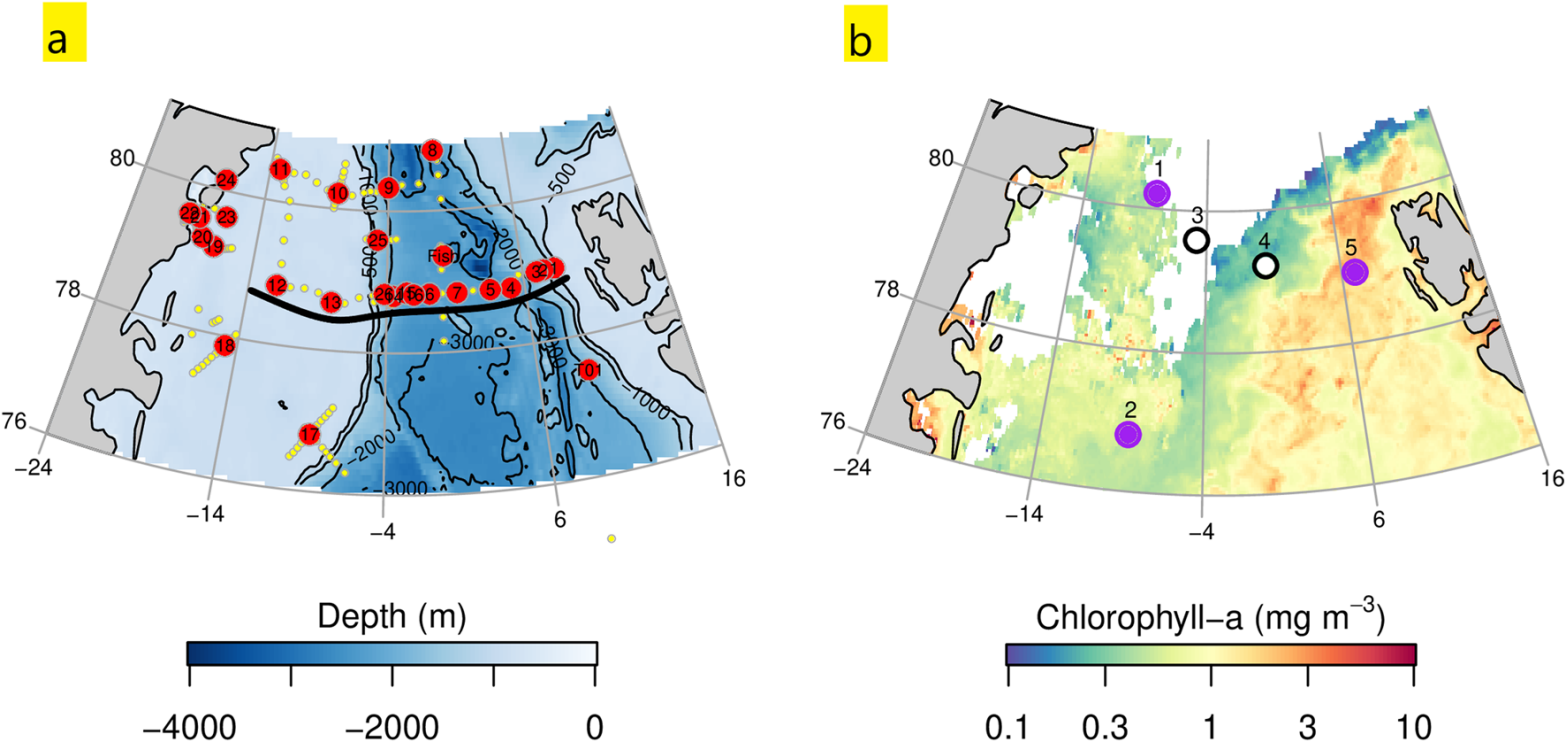
(**a**) Bathymetry and location of ucCTD stations sampled in the Fram Strait and on the NE Greenland Shelf. Black line highlights the section from which depth profiles are obtained (Fig. 3). (**b**) Surface chlorophyll-a concentrations (August 2016 average) obtained from MODIS-Aqua (NASA GES DISC)^105^. Dots indicate location and outcome of nutrient addition bioassay experiments (E1–E5): Purple = N limitation; white = no response to N, Fe or dust addition. White area indicates sea-ice cover. Figure generated using Matlab software version 9.6.0. (R2019a, Natick, Massachusetts: The MathWorks Inc., 2019).

**Figure 2:**
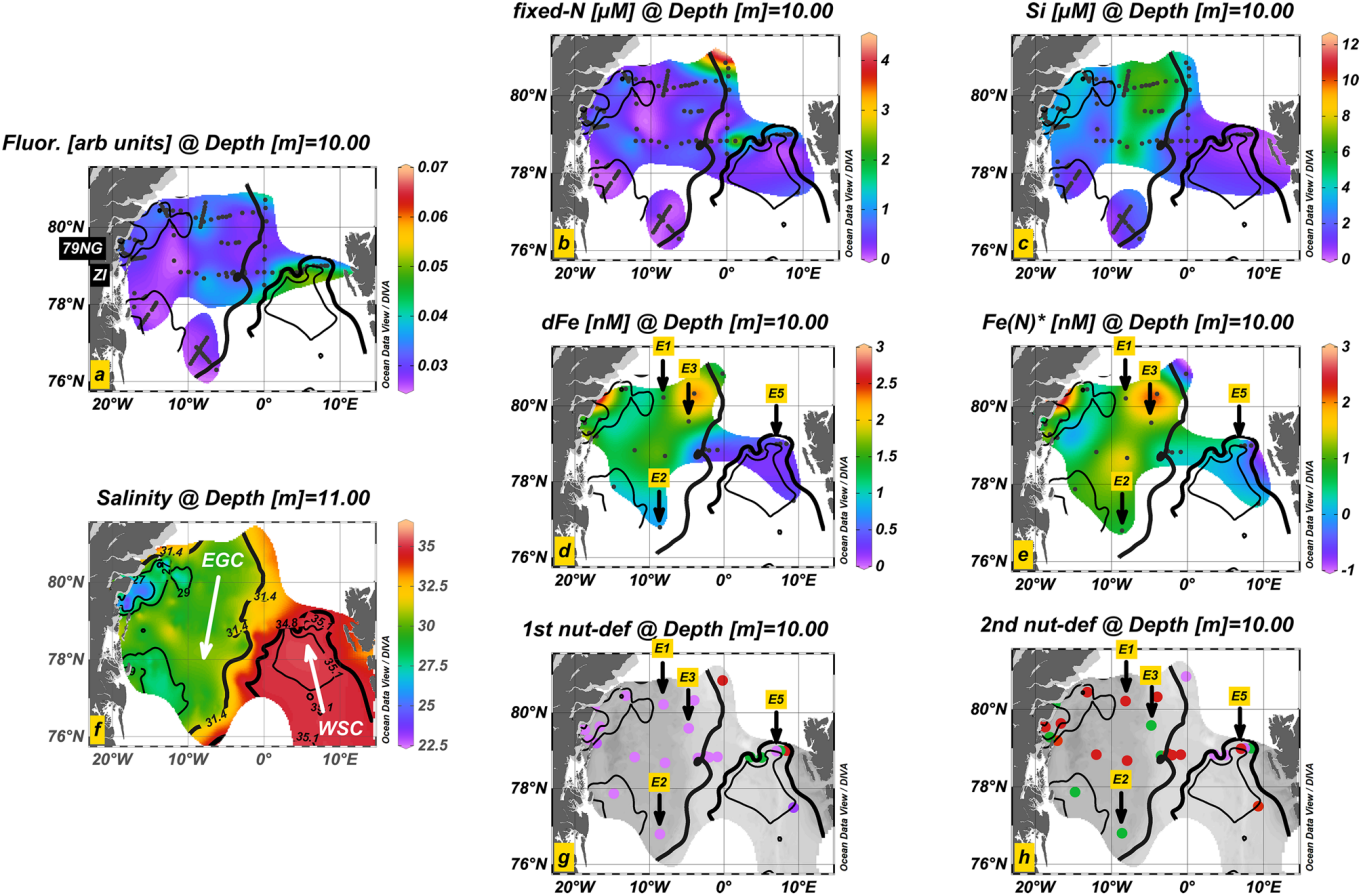
Salinity and nutrient distributions at 10–11 m depths in the Fram Strait region. (**a**) Salinity, (**b**) fixed N, (**c**) Si, (**d**) dFe, (**e**) Fe(N)* nutrient deficiency, and (**f**) fluorescence. Primary (**g**) and secondary (**h**) nutrient deficiency after Moore^35^: Fixed N (purple), dFe (red) and Si (green). Black dots indicate the sampling locations (all 10 m depth). Black arrows indicate sites of bioassay experiments (excl. E4). The location of Nioghalvfjerdsbrae (79NG) and Zachariæ Isstrøm (ZI) are depicted in ‘a’. Isohalines (contours, 11 m) distinguish the Polar Surface Water (S < 31.4, bold) of the southward-directed East Greenland Current (EGC, S < 34.8, < 0°E) from Intermediate Water (34.8–35.1) and Atlantic Water (S > 35.1, thin black) of the northward-flowing West Spitsbergen Current (WSC). Figure made using Ocean Data View software based on DIVA gridding calculations (Schlitzer, R., Ocean Data View, version 5.2.1, https://odv.awi.de, 2020).

The Fram Strait region (76–82°N, 20°W–15°E) forms the northern boundary of the Greenland Sea and is the major gateway for water exchange between the Arctic and North Atlantic Oceans (Fig. 1)^20–22^. The region features two surface currents: (i) the West Spitsbergen Current (WSC) with warm and saline Atlantic Water (4 °C, S > 35.10) at its core and a less saline surface layer (< 65 m) (Fig. 2a), flowing northwards from the Greenland Sea towards the Arctic Ocean^22,23^; and (ii) the southward flowing East Greenland Current (EGC), which originates in the Arctic Ocean, includes a contribution of NE Greenland Shelf waters, and constitutes low-salinity Polar Surface Water (S < 31.40) and more-saline sub-surface waters (S < 34.80) below 50–250 m^23–25^. About half of the Fram Strait region is covered by seasonal sea ice, leading to strong year-round light limitation of phytoplankton growth^24,26,27^. However, the extent of Arctic sea ice has diminished considerably over the past three decades (~ 40%, 1979–2018) (data from the Norwegian Polar Institute, 2020) prolonging the areal extent and duration of enhanced light availability for phytoplankton growth. At the same time, Fram Strait has experienced surface freshening that has driven enhanced water column stratification^28,29^, potentially linked to variations in the advection and recirculation of Atlantic-derived waters^16,30,31^. It remains unclear how these on-going physical changes are affecting nutrient supply to surface waters and thereby primary production in the region.

The aim of this study was to assess the factors that currently regulate summertime phytoplankton growth in the Fram Strait region and are hence key to predicting responses of this system to future climate change. We therefore (i) performed a detailed investigation into nutrient and micronutrient concentration distributions and stoichiometry across Fram Strait as part of the GEOTRACES GN05 cruise, and (ii) conducted nutrient addition bioassay experiments as direct assessments of spatial patterns in limiting (micro-)nutrients.

## Methods

### General

Samples were collected on the RV Polarstern (PS100) as part of the GEOTRACES programme (section GN05). The cruise investigated Fram Strait (> 500 m bottom depth) and the adjacent NE Greenland Shelf (< 500 m, together the ‘Fram Strait region’) in the period 23^rd^ July to 1^st^ September 2016 (boreal summer), which corresponded to the peak melt season. In total, 207 stations were occupied. The water column was sampled with a stainless steel CTD-rosette frame (equipped with SEA-BIRD SBE 911plus) and a powder-coated aluminium GEOTRACES CTD-rosette frame (Seabird, equipped with SBE 911, hereafter ‘ucCTD’). All stations were sampled for macronutrients silicic acid (Si(OH)_4_, hereafter Si), phosphate (PO_4_), ammonium (NH_4_), nitrite (NO_2_), and nitrate (NO_3_) from the stainless steel CTD. Here we refer to the sum of NH_4_, NO_2_ and NO_3_ as fixed N. Trace metal micronutrients manganese (Mn), Fe, cobalt (Co), nickel (Ni), copper (Cu) and zinc (Zn) were sampled from the ucCTD at 26 stations (Fig. 1a). Near-surface temperature and salinity were recorded continuously during the cruise from the ship’s thermosalinograph (11 m intake depth). The stainless steel CTD was additionally equipped with a fluorometer (WET Labs ECO AFL/FL).

### Nutrient and trace metal analyses

Unfiltered macronutrient samples (NO_2_, NO_3_, PO_4_, Si, NH_4_) from the upper 200 m were analysed on board using a QuAAtro autoanalyser as per Grasshoff et al.^32^, modified according to the methods provided by the manufacturer (Seal, Alliance). At all other depths filtered samples (0.2 µm) were frozen and run at AWI following an identical procedure. Measurements were validated against the certified reference material NMIJ CRM 7602a provided by the National Metrology Institute of Japan (see data publication).

Trace metal samples were collected in 24 × 12 L GoFlo bottles (Ocean Test Equipment, OTE) following GEOTRACES sampling and sample handling protocols^33^. Briefly, GoFlo bottles were transferred into an over-pressurized clean room (class 100) where water samples were filtered (Acropak 0.8/0.2 µm pore size) into pre-cleaned 125 mL low density polyethylene (LDPE) bottles with slight overpressure provided by filtered nitrogen gas (99.999%, AlphaGaz). One additional surface (~ 2 m) seawater sample was collected in mid Fram Strait using a tow-fish sampling system with suction provided by a Teflon diaphragm pump (Almatec A15). Samples were acidified to pH 1.9 with 180 µL HCl (UpA, ROMIL) and shipped to GEOMAR to determine dissolved concentrations of Mn (dMn), Fe (dFe), Co (dCo), Ni (dNi), Cu (dCu) and Zn (dZn). Trace metal samples were measured via high-resolution inductively coupled plasma-mass spectrometry (HR-ICP-MS) after pre-concentration following the method of Rapp et al.^34^. Briefly, 15 mL sample aliquots were pre-concentrated using an automated SeaFAST system (SC-4 DX SeaFAST pico; ESI). All reagents for SeaFAST were prepared in de-ionised water (> 18.2 MΩ cm^−1^; Milli-Q, Millipore). Single-distilled sub-boiled HNO_3_ (SpA grade, Romil) was used for sample elution. Ammonium acetate buffer (pH 8.5) was prepared from glacial acetic acid and ammonium hydroxide (Optima, Fisher Scientific). The tenfold pre-concentrated samples were analysed by HR-ICP-MS (Thermo Fisher Element XR) with quantification via isotope dilution for dFe, dNi, dCu and dZn (^57^Fe-, ^62^Ni-, ^65^Cu- and ^68^Zn-spike), and standard addition for dMn and dCo (every 10th sample). Measurements were checked against SAFe S reference material (Bruland Research Lab, University of California Santa Cruz) (see data publication).

The deficiency of each nutrient element relative to N was calculated using assumed average phytoplankton stoichiometry (C_7.75_N_1_Si_1_P_0.0625_)_1000_Fe_0.469_Mn_0.175_Ni_0.0625_Zn_0.05_Cu_0.024_Co_0.012_ (extended Redfield ratio; Ref.^35^), measured dissolved nutrient concentrations, and the following equation:

1 $${X}_{N}^{*}={\mathrm{\rm X}}_{\mathrm{dis}}-{R}_{X:N}\bullet \mathrm{ N}$$

where ${\mathrm{\rm X}}_{\mathrm{dis}}$ is the concentration of dissolved nutrient X, N is the concentration of fixed N, and ${R}_{X:N}$ is the assumed phytoplankton requirement for nutrient X relative to N^4,35^. Positive values of ${X}_{N}^{*}$ suggest nutrient Χ is in excess relative to fixed N, while negative ${X}_{N}^{*}$ values indicate nutrient X is deficient relative to fixed N^35^. Following Moore^35^ we then ranked the deficiencies for each nutrient at each site using:

2 $${X}_{N}^{^{\prime}}= \frac{{\mathrm{\rm X}}_{\mathrm{dis}}}{N \bullet {R}_{X:N}}$$

The nutrient with minimum ${X}_{N}^{^{\prime}}$ is the most deficient.

### Incubation experiments

Incubation experiments were conducted using seawater collected from the OTE Go Flo bottles (~ 10 m depth; Experiments E1, E2, E3 and E5) or the tow-fish (2 m depth; E4) following procedures described in Browning et al*.*^36^. All sampling and sample handling was carried out on-board using trace metal clean techniques under a laminar flow hood. Eighteen trace metal clean, 1 L polycarbonate bottles (Nalgene) were filled with unfiltered seawater. Three bottles were subsampled immediately for assessment of initial conditions. Of the remaining fifteen bottles, three were spiked with fixed N (1 µM nitrate + 1 µM ammonium), three with Fe (2 nM FeCl_3_), three with fixed N + Fe, and three with ~ 10 mg volcanic dust from the Eyjafjallajökull volcanic eruption in 2010 (same sample as described in Achterberg et al*.*^37^). The fixed N spike solutions were previously passed through a column of Chelex-100 to remove contaminating trace metals. Three bottles were sealed immediately with no amendment (controls). The lids were sealed with Parafilm and the bottles double-bagged before being placed in an on-deck incubator, which was filled and continuously replenished with near-surface seawater from the ship’s underway supply. The incubator was shaded with blue screening (Lee Filters ‘Blue Lagoon’) to yield incubator light intensities of ~ 35% of surface values. Samples were incubated for 72 h. Both initial (t = 0 h) and incubated (t = 72 h) samples were subsampled for chlorophyll-a via filtration of 100 mL sample onto a glass fibre filter (GFF, 0.7 µm nominal pore size, 25 mm diameter, Macherey–Nagel), extracted in the dark in 10 mL 90% acetone at − 20 °C (~ 12 h), then measured using a calibrated Turner Designs Trilogy fluorometer^38^.

At experimental start sites, samples were collected for determination of macronutrient and trace metal concentrations and phytoplankton pigment concentrations. Samples for phytoplankton pigments were collected by filtration of 2–4 L of seawater onto GFF filters and stored at − 80 °C. Upon return to the home laboratory, samples were analysed following van Heukelem and Thomas^39^. Pigments were extracted in 90% acetone in plastic vials using glass beads in a cell mill, centrifuged (10 min, 5,200 rpm, 4 °C), and then the supernatant was filtered through PTFE filters (0.2 µm pore size, VWR International) and analysed by reverse-phase HPLC (Dionex UltiMate 3,000 LC system, Thermo Scientific). Pigment standards were acquired from Sigma-Aldrich (USA) and the International Agency for ^14^C Determination (Denmark). Pigment compositions were converted to the approximate contribution of different phytoplankton types to total chlorophyll-a using CHEMTAX^40^ with starting pigment ratios from Coupel et al.^41^.

## Results and discussion

### Nutrient distributions

Fixed N displayed an east to west gradient in surface waters (10 m depth) (Fig. 2b). Maximum surface concentrations were found in the WSC (0.7 ± 1.0 µM, mean value ± standard deviation) and at the northern-most stations in Fram Strait (1.4 ± 1.1 µM, 80.2–80.8°N/0–4°W). Elsewhere, relatively low fixed N concentrations were observed (0.3 ± 0.3 µM). Surface Si showed a north-to-south gradient within the eastern part of the EGC (Fig. 2c). Highest Si concentrations were found at higher latitudes (4.2 ± 1.6 µM at all EGC stations > 79°N), whereas lower Si concentrations were found at the NE Greenland Shelf break to the south (1.2 ± 0.5 µM at all EGC stations < 77°N). The WSC was depleted in Si (0.7 ± 0.7 µM), which contrasted to the EGC with elevated Si (3.5 ± 2.9 µM for all stations in the EGC). Surface dFe displayed maxima on the shelf at glaciated stations close to the Greenlandic coast (Fig. 2d; S19–S24, 1.8 ± 1.0 nM). DFe concentrations within the EGC (1.3 ± 0.6 nM) exceeded those in the WSC (0.4 ± 0.1 nM) by a factor of 3–4. In Fram Strait, dFe showed a similar trend to Si and was elevated at the two northern-most stations in its central and western parts (S8, 1.3 nM; S9, 2.5 nM), in contrast to the observed minimum dFe in the central WSC in the eastern Fram Strait (S4, 0.2 nM). Collectively, dFe and Si both showed elevated concentrations at low salinity (Fig. S1), which was most evident at the eastern boundary of the Polar Surface Water (0–10°W). In contrast to dFe and Si, no clear special pattern in surface fixed N concentrations relative to salinity was observed across the study region.

**Figure 3:**
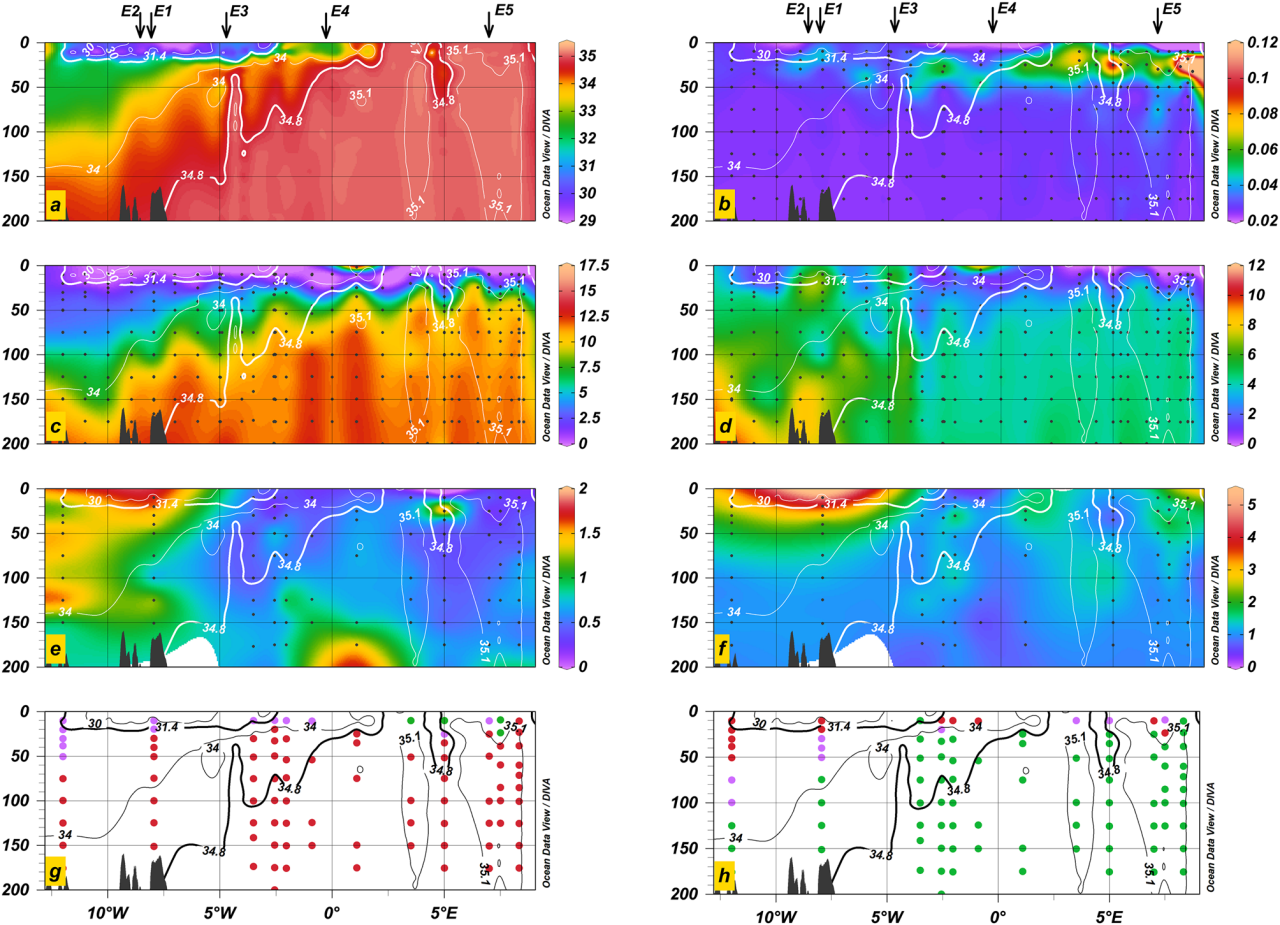
Fram Strait sections (79°N) of (**a**) salinity, (**b**) fluorescence (arb. units), (**c**) fixed N (µM), (**d**) Si (µM), (**e**) dFe (nM) and (**f**) dissolved manganese (nM), (**g**,**h**) derived primary and secondary nutrient deficiency (see Eq. (2)). Fixed N (purple), dFe (red), Si (green) and dCo (orange). Black arrows (top panel) indicate the longitudinal positions of the nutrient addition bioassay experiments. The location of the presented section is shown in Fig. 1a. Isohalines (contours) indicate the presence of Polar Surface Waters (S < 31.4, bold) as part of the East Greenland Current (S = 34.8, bold), Intermediate Water (34.8 < S < 35.1) and core Atlantic Water (S > 35.1). Black dots in b-f indicate the sample depths. Figure made using Ocean Data View software based on DIVA gridding calculations (Schlitzer, R., Ocean Data View, version 5.2.1, https://odv.awi.de, 2020) and RTopo-2.0.1 bedrock topography^106^.

Concentrations of N and Si uniformly increased with depth, whereas depth profiles of dFe showed much greater spatial variability (Fig. 3c–e). Water column distributions of fixed N, Si and dFe in the WSC were distinctly different from the EGC. In the WSC, fixed N and Si were depleted (< 5 µM) above ~ 25 m and ~ 450 m, respectively. In the EGC, fixed N remained depleted to greater depths nearer Greenland (21–148 m), and mean concentrations were lower (6.1 µM ± 4.3 µM for salinities < 34.8, corresponding to depths < 20–454 m) compared to the WSC (9.4 µM ± 3.7 µM in the water column at salinities > 35.1, corresponding to depths < 71–261 m). Si on the contrary was enriched in the EGC (5.4 µM ± 2.3 µM) relative to the WSC (3.7 µM ± 1.3 µM). The distribution of Si at depth followed the spatial pattern found in surface concentrations, showing a north-to-south gradient in the water column of the EGC. Elevated concentrations were observed at higher latitudes (all EGC stations > 79°N), near the glacial termini of Zachariæ Isstrøm and Nioghalvfjerdsbrae (6.3 µM ± 3.2 µM) and at the NE Greenland Shelf break (5.8 µM ± 1.3 µM, EGC stations 4–8°W) compared to lower latitudes (3.8 ± 1.8 µM, EGC stations < 77°N). Enhanced Si concentrations (> 10 µM) on the inner-Greenlandic shelf at depth likely reflect elevated benthic inputs^42^. Mean EGC dFe concentrations (1.1 nM ± 0.5 nM) were ~ twofold elevated over WSC values (0.5 nM ± 0.2 nM). Depth profiles of dFe showed sporadic enrichment (> 1 nM) that coincided with dMn (Fig. 3f) at multiple depths near Svalbard’s continental slope, likely derived from sedimentary inputs as has been observed elsewhere in the high latitude North Atlantic^43,44^, and in central Fram Strait. Enrichment of dFe in the EGC was more sporadic than for Si, likely as a consequence of rapid scavenging of dFe sources along the Greenlandic shelf^45,46^. However, as for Si, dFe displayed maxima in the water column off Zachariæ Isstrøm and Nioghalvfjerdsbrae (S20, S22, S24, 1.5 nM ± 0.6 nM) and near the NE Greenland shelf break (S9, S10, S12, S13, 1.2 nM ± 0.5 nM) indicating similar shelf-associated Si and dFe sources.

A similarly extensive survey of macronutrient distributions in the Fram Strait region was previously conducted in July–August 2008. Our observations of fixed N (Jul–Aug 2016) agree well with these previous measurements, showing surface depletion (< 5 µM) increasing in depth from the WSC (16–42 m) to the EGC (12–129 m)^47,48^. Mean fixed N concentrations within the EGC (6.1 ± 4.3 µM in all EGC stations S > 34.8) were comparable to observations made in 2008 (5.3 ± 4.1 µM), but fixed N was somewhat higher in the WSC (9.4 ± 3.7 µM in the water column at salinities > 35.1) compared to 2008 (5.7 ± 4.2 µM), possibly related to increased advection and/or recirculation of Atlantic Water to the Fram Strait region in 2016^30,31^. Surface fixed N in the WSC (0.7 ± 1.0 µM) was similar to 2008 (0.9 ± 1.2 µM), and falls between observations of the upstream Irminger Basin (2.3 ± 1.7 µM, Jul–Aug 2010) and the downstream Arctic Ocean (Aug 2014) where complete depletion of surface Atlantic Water fixed N (< 0.02 µM) is evident^49,50^. Atlantic Water is thus an essential source of fixed N to the Fram Strait region^51^. The distribution of Si showed an east-to-west gradient in 2008 similar to 2016, with elevated concentrations in the EGC at high latitudes (5.2 ± 2.2 µM, in all EGC stations > 79°N) relative to lower latitudes (4.6 ± 2.2 µM in all EGC stations at 77°N). This north–south gradient was most pronounced for surface Si (2.6 ± 1.2 µM, > 79°N; 1.4 ± 0.2 µM at 77°N), and is further evident when considering Si concentrations further north from within the Transpolar Drift (12.9 ± 0.6 µM, Sep 2015)^52,53^, and further south from the EGC bordering the Irminger Basin (1.4 ± 0.7 µM at 20 m, 58.1–60.0°N/35.0–42.7°E, Jul–Aug 2010)^50^. DFe shows the same north–south trend, with concentrations > twofold elevated in the surface Transpolar Drift (2.9 ± 1.3 nM)^53,54^, and an order of magnitude further depleted in the Iceland (0.1 ± 0.1 nM) and Irminger Basins (0.1 ± 0.2 nM)^9,50^. EGC dFe and Si may thus share a common regional source in the Arctic Ocean, and may themselves in turn be a source to the Greenland Sea phytoplankton communities.

### Phytoplankton abundance and community structure

**Figure 4:**
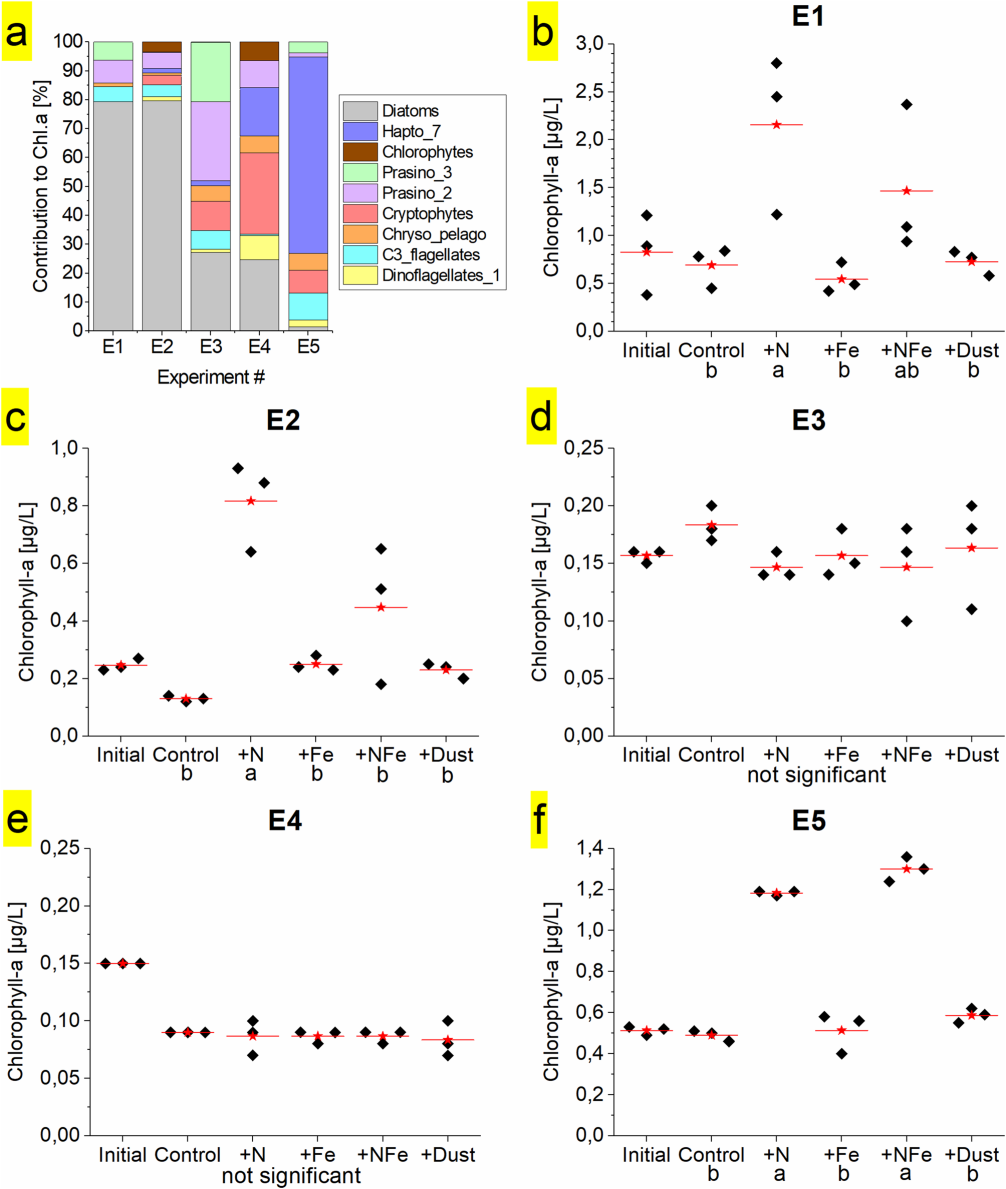
Phytoplankton community composition and responses to nutrient supply at incubation sites. (**a**) Phytoplankton community composition at bioassay experiment water collection sites as determined by pigment analysis and CHEMTAX. (**b**–**f**) Chlorophyll-a responses to nutrient additions. Red symbols show the mean chlorophyll concentration and black symbols indicate the individual replicates. Treatments labelled with the same letter have statistically indistinguishable means (ANOVA followed by a Tukey Honest Significant Difference test, *P* ≤ 0.05, n = 3). Figure generated using OriginPro software, version 9.1.0 (OriginLab Corporation, Northampton, MA, USA).

Satellite-derived surface chlorophyll-a concentrations showed an east-to-west transition in phytoplankton standing stocks in August 2016, with minima in Central and Western Fram Strait, and maxima in Atlantic Waters northwest of Svalbard (Fig. 1b). Laboratory-determined chlorophyll-a concentrations matched the satellite-derived trend for the WSC (0.5 µg L^−1^), Western and Central Fram Strait (0.2 µg L^−1^), yet indicated highest phytoplankton concentrations in the EGC on the outer NE Greenland Shelf (0.8 µg L^−1^) (Table 1). Measured pigment assemblages and associated CHEMTAX data analysis suggested an east to west transition in surface phytoplankton community composition that correlated with the region’s nutrient and current regimes (Figs. 2 and 4a). On the Si- and dFe-replete NE Greenland shelf, diatoms were the dominant phytoplankton group (E1–E2, 80% of chlorophyll-a was attributable to diatoms). A similar dominance has been observed elsewhere in coastal Greenlandic waters which remain Si-replete post-spring bloom^55,56^. The diatom contribution decreased alongside surface Si and dFe concentrations towards western Fram Strait (E3, 27%) and central Fram Strait (E4, 25%) and was lowest in eastern Fram Strait (E5, 1%). The Eastern Fram Strait phytoplankton composition was dominated by haptophytes (E5, 68%) matching earlier observations in the Si-depleted summer WSC^57^. Haptophyte contribution declined considerably to central Fram Strait (E4, 17%) and was almost absent at western Fram Strait (E3, 2%) and on the NE Greenland Shelf (E1–E2, 0–1%). Whilst both the NE Greenland shelf and eastern Fram Strait showed dominance of either diatoms or haptophytes, stations located in western (E3) and central (E4) Fram Strait exhibited mixed assemblages of phytoplankton including enhanced contributions of cryptophytes (10% and 28% respectively), and prasinophyceae (31% and 9%).

**Table 1:** Initial conditions of bioassay experiments, and chlorophyll-a biomass response to nutrient amendments.

		E1	E2	E3	E4	E5
Lat	°N	80.22	76.80	79.59	79.36	78.99
Long	°E	− 8.14	− 8.61	− 4.77	0.23	6.99
T_pot_	°C	− 1.3	0.1	− 1.6	2.7^Ŧ^	8.8
Sal	PSU	29.97	30.62	30.03	32.67^Ŧ^	35.07
MLD^§^	m	12	10	14	/	14
NO_3_	µM	< 0.02	< 0.02	0.5	12.3	0.3
PO_4_		0.4	0.3	0.3	1.0	0.1
Si		4.4	1.1	4.4	7.4	< 0.03
NH_4_		< 0.05	< 0.05	0.5	1.6	< 0.05
dCo	pM	202	148	164	114*	40
dFe	nM	1.3	0.8	1.8	1.3*	0.4
dMn		5.0	4.2	5.6	2.8*	1.5
dNi		6.2	4.7	5.9	4.4*	3.1
dCu		4.8	3.7	4.5	2.9*	1.3
dZn		1.8	/	1.8	0.9*	0.8
Fe_N_* initial	nM	1.3	0.8	1.3	− 5.3*	0.3
Initial	Chl.a in mg/m^3^	0.82	0.25	0.16	0.15	0.51
Control		0.69	0.13	0.18	0.09	0.49
+N		2.16	0.82	0.15	0.09	1.18
+Fe		0.54	0.25	0.16	0.09	0.51
+N + Fe		1.47	0.45	0.15	0.09	1.30
+Dust		0.73	0.23	0.16	0.08	0.59
N-to-initial	%	313	628	80	58	241
(N + Fe)-to-initial		213	344	80	96	265

^§^Surface mixed layer depth (MLD) defined by threshold value of 0.03 kg/m3 difference to surface reference density (10 m depth).

^Ŧ^Values obtained from ship-based thermosalinograph (11 m depth).

*Trace metal concentrations taken from station S8.

### Nutrient limitation

Phytoplankton responses to nutrient amendment were assessed via changes in chlorophyll-a concentrations, which likely reflects contributions from both bulk phytoplankton biomass changes and enhances in chlorophyll-a: carbon ratios following supply of limiting nutrient(s)^58,59^. Chlorophyll-a concentrations increased significantly relative to untreated controls following addition of fixed N in Experiments 1, 2, and 5 and did not vary following supply of any nutrient combination in Experiments 3 and 4 (Fig. 4b–f). For Experiments 1, 2, and 5, relative chlorophyll-a increases following N supply were larger in experiments around the EGC shelf break (mean increases of + 313% in E1; + 628% in E2) than in the WSC in eastern Fram Strait (+ 241% in E5). Our observations of significant chlorophyll-a increases following N supply in these experiments suggested that fixed N was the primary summertime limiting nutrient through these parts of the Fram Strait region during our cruise occupation. These observations are consistent with prior work on the NE Greenland Shelf^60^ and elsewhere in the Arctic Ocean^11,13,61–63^. Although not statistically significant, small additional enhancements in chlorophyll-a concentrations were observed in the Experiment 5 N + Fe treatment over + N alone (+ 265% in comparison to + 241% for N addition alone), suggesting that Fe was potentially a secondary limiting nutrient after N in the surface WSC. The absence of any significant chlorophyll-a enhancements in response to supply of the volcanic ash dust in any of the experiments suggested that the dust was not a significant source of fixed N, as anticipated based on the results of previous bioassays and leaching experiments with this ash sample^37^.

No statistically significant responses to any of the supplied nutrient combinations relative to unamended controls were found in Experiments 3 and 4, located in the western and central Fram Straight respectively. This could either be a result of (i) limitation by an alternative resource (light or another nutrient); (ii) grazer regulation of the phytoplankton community, maintaining phytoplankton standing stocks despite enhanced specific growth rates following nutrient supply (e.g., Mann and Chisholm^64^); or (iii) restricted net phytoplankton community growth over the experimental duration (72 h) due to low starting biomass concentrations and/or cold temperatures^65^. Our results do not allow us to unambiguously distinguish between these; however, ancillary observations allow identification of the most probable factors. Light limitation of the ambient phytoplankton community at these sites was unlikely, as surface mixed layers were shallow (14 m at Experiment 3, Table 1), irradiances were elevated (E3: up to 395 W m^−2^, E4: up to 478 W m^−2^), and day lengths long (19–24 h). Furthermore, in-situ light limitation, if present, would likely be at least partially relieved following incubation on-deck, leading to chlorophyll-a increases in control samples^66,67^, which were not observed. (Co-)limitation of phytoplankton growth by another element cannot be ruled out, however concentrations of all measured nutrients were elevated, arguing against this (Table 1). In addition, the absence of any response following the volcanic dust addition, which likely supplied a range of trace metal micronutrients alongside Fe^37^, suggested that non-Fe trace elements were also not primary limiting nutrients. Slow, nutrient-replete growth, low starting phytoplankton concentrations, in combination with top-down grazer regulation therefore appears the most likely cause of the lack of net community growth response in these two experiments.

### Nutrient deficiency

The nutrient concentrations in seawater relative to typical phytoplankton requirements provide the capacity to predict which nutrient becomes limiting to phytoplankton growth^35^. Either fixed N or Fe was calculated to be the most deficient nutrient in surface waters of the Fram Strait region (that is, had lowest concentrations in seawater taking into account assumed-average phytoplankton requirements; Eq. (2), Fig. 2). An east-to-west transition was found in the deficiency of Fe relative to N (Fe_N_*). Surface waters of the EGC, where fixed N limitation was observed, showed positive Fe_N_* (mean 1.1 ± 0.9 nM, range − 0.3 to 3.2 nM), which increased towards the Greenlandic coast and the glaciated Zachariæ Isstrøm (S20, 1.7 nM) and Nioghalvfjerdsbrae Glacier (S21, 1.2 nM; S24, 3.3 nM) (Fig. 3d). Interestingly, the station closest to Nioghalvfjerdsbrae exhibited the EGC minimum in Fe_N_* (-0.3 nM, S22), likely a result of low dFe/ low fixed N meltwater discharge in combination with dFe scavenging removal^14,45^. The surface WSC showed lower Fe_N_* (0.1 ± 0.4 nM, -0.7–0.3 nM), with decreasing values towards the Svalbard shelf break. The station closest to Svalbard (S1) had lowest Fe_N_* (-0.7 nM) at low residual dFe (0.3 nM), suggesting phytoplankton may have may have been approaching a situation of co-limitation by Fe and N^10,36,68^. This is in agreement with the results of Experiment 5, where an enhanced chlorophyll-a response to Fe + N amendment over + N alone was apparent. In addition, some surface waters of the WSC also showed Si deficiency relative to fixed N, suggesting that this nutrient might be (co-)limiting for Si-requiring diatoms in this location and potentially, alongside low N and Fe levels, partly explaining their absence in these waters (Figs. 3g and 4). Elsewhere, Si was generally in excess relative to N.

Subsurface (> 10 m) waters were always Fe deficient relative to fixed N throughout the central Fram Strait region (Fig. 3g), presumably caused by remineralisation-driven increases in macronutrient concentrations, which are not reflected in dFe as a result of the high particle reactivity and scavenging removal of this element^35,69^. Sub-surface N-deficiency was only observed on the NE Greenland Shelf where the nitracline was deeper, suggested to be a consequence of release of low N glacial meltwater, or more generally strong stratification driven by freshwater. Making the assumption of a phytoplankton requirement for Si, this element was typically calculated as the second most deficient nutrient throughout subsurface waters (Fig. 3h). Repeating the analysis with Si excluded instead showed widespread secondary deficiency by dissolved Co.

### Factors controlling nutrient limitation in Fram Strait

Like other marine regions, the drivers of phytoplankton nutrient limitation patterns strongly depend on the relative supply: demand ratio for each nutrient^4^. In order to categorize measured nutrient concentrations in terms of limitation potential, we assumed fixed elemental requirements for phytoplankton. The potential for high variability in elemental quotas of phytoplankton, particularly under reduced competition that exists in monoculture, has been reported^70^. However, the success of nutrient deficiency calculations in correctly predicting the identity of the growth limiting nutrient in the ocean (i.e., following direct experimental determination or via molecular nutrient stress signatures) argues for a relatively conserved, community-level elemental stoichiometry in natural phytoplankton communities^4,36,71^. Changes in nutrient limitation patterns, and in particular the presence of N versus Fe limitation, or the development of co-limitation, will therefore likely have a strong dependence on altered supply rates and ratios of these nutrients to the surface ocean^36,72^.

In this respect, the southward flowing EGC and the northward flowing WSC are distinctly different in physical characteristics, nutrient content, and thereby nutrient supply to surface waters. The EGC was colder and fresher, with regionally-averaged concentrations having lower fixed N but 2–fourfold higher Si and dFe compared to the WSC (Table 2). Intermediate Waters, defining the boundary between these two water masses, showed values between these end members, which may have been derived from the convergence of the EGC and WSC^23,25,73^. Elevated dFe and Si in the EGC was likely a result of locally-derived freshwater and southward transport in the Transpolar Drift, itself fuelled by riverine discharge to the Eurasian Arctic Shelf^45,74,75^. Conversely, these sources supply low amounts of fixed N^14,52,53^. In addition to the low N:P ratio of Pacific water, which is advected across the Arctic and exits via Fram Strait^15,76^, this may be a contributing factor to the observed primary N limitation of the phytoplankton community in this region (Figs. 3 and 4). In contrast, North Atlantic source waters supplying the WSC are depleted in Fe and Si, alongside N, which leads to conditions approaching serial N-Fe limitation (and potential co-limitation by Si for diatoms) in the eastern Fram Straight^10,67,77^. Further north, a branch of the WSC continues to flow into the SE Nansen Basin, where – off-axis to the dFe-rich Transpolar Drift—Fe deficiency (i.e. Fe_N_* < 0) has also been reported^19,74^.

**Table 2:** Region-averaged physical characteristics and nutrient concentrations (mean value ± standard deviation) of the East Greenland Current (EGC), West Spitsbergen Current (WSC), and Intermediate (IW) surface (10 m) waters.

		EGC	IW	WSC
T_pot_	°C	− 0.5 ± 0.7	− 0.4 ± 2.2	8.3 ± 0.7
Sal	PSU	30.37 ± 0.78	32.43 ± 1.97	35.02 ± 0.06
Fixed N	µM	0.3 ± 0.5	0.6 ± 0.6	0.7 ± 1.0
dFe	nM	1.5 ± 0.8	0.8 ± 0.5	0.4 ± 0.1
Si	µM	3.5 ± 2.9	2.3 ± 1.2	0.7 ± 0.7

Prior work has shown that southward transport of N associated with the EGC (124 ± 22 Tg yr^−1^) is almost balanced by northward transport associated with the WSC (92 ± 17 Gg yr^−1^)^51^. Surface Waters of the Fram Strait region were N depleted (0.5 ± 1.2 µM), but deeper waters below the mixed layer hosted enhanced fixed N concentrations (15–500 m, 8.4 ± 4.1 µM) (Fig. 3). Summer mixed layer depths in Fram Strait are shallow (10–23 m measured on the PS100 cruise, Table 1) and deep-water reservoirs are thus unlikely a substantial source of fixed N to the surface ocean at this time^49^. Conversely, in winter, deep mixing to depths > 100 m below the nutrient-depleted surface layer (10–25 m), replenishes surface waters of Fram Strait region with N^15,78^. Rates of surface WSC fixed N replenishment may be an order of magnitude greater during autumn/winter than summer^49,79^, making deep winter mixing likely the dominant factor controlling the extent of annual new primary production in the Fram Strait region^80^. Deep-water re-supply of surface Fe is more restricted, due to deeper ferriclines that are observed to depths > 150 m in the WSC of Fram Strait (Fig. S2). Therefore to account for near-complete drawdown of fixed N between winter and summer in the WSC, Fe must have additional supply routes (assuming that seasonally or regionally enhanced regeneration/recycling of Fe cannot fully account for such drawdown^81^). Aside from transient, low-frequency events such as volcanic eruptions^37^ or agricultural and forest fires^82^, atmospheric deposition of Fe across the Arctic is reportedly low^83,84^. This suggests a dominance of sediment supplied Fe from shallow bathymetry to the south (i.e. coastal Greenland and Svalbard) and supply of Arctic-derived Fe by the Transpolar Drift to the EGC waters (offshore Fram Strait region)^75^. The importance of sedimentary dFe supply to Fram Strait region was also indicated from the distribution of dissolved Mn (Fig. 3f), which derives primarily from sediments and shows maxima in (i) the surface EGC where the influence of the Transpolar Drift is expected^85^, (ii) near the glacier termini on the NE Greenland Shelf, and (iii) on the continental slopes of Svalbard^86–88^.

### Future perspectives

Our finding of widespread N deficiency in nutrient concentration fields alongside direct experimental evidence for N limitation points towards the importance of variability in N sources in regulating summertime phytoplankton productivity in Fram Strait. Consistent with prior studies, we corroborate that northward advection of Atlantic-derived fixed N is essential to sustain primary production in the N-depleted western Fram Strait and on the NE Greenland Shelf^14,24,60,79^. Accordingly, we suggest that the future areal and seasonal extent of primary production in Fram Strait region might be strongly impacted by changes in the lateral advection of fixed N via the WSC, in addition to an earlier onset of phytoplankton growth resulting from reduced ice cover^79,89^. As an Arctic outflow region, Fram Strait is clearly strongly affected by processes occurring within the Arctic. Broad-scale changes to Arctic inflow, sea-ice cover, lateral advection via the Transpolar Drift and wind driven mixing will all have downstream effects on nutrient stoichiometry in Fram Strait^80,90,91^. How lateral and vertical nutrient supply will change in the future Arctic as a result of on-going changes to sea-ice retreat and stratification remains uncertain, as neither lateral transport of nutrients in the Transpolar Drift, or vertical turbulent supply of nutrients during summer are well quantified^13,75,92^.

Surface primary production in the Arctic Ocean due to increased summertime light availability has increased during the past few decades^3,93^. Second to light limitation, Arctic Ocean net primary production seems foremost limited by the availability of fixed N^15,80^ and may at some point hinder future increases in productivity, although changes in nutrient supply ratios and/or phytoplankton species composition may result in other nutrients such as Si, PO_4_ or dFe to become limiting factors as well^11,94^. In the future, the Arctic Ocean is projected to have less ice coverage and become fresher and more stratified, which would reduce winter mixing depth and the associated supply of deep water nutrients^8,95^. Yet, conversely sea-ice loss also facilitates increased wind driven mixing, which is thought to have enhanced autumn productivity in some Arctic regions^96,97^, and may also offset nutrient limitation caused by water column stratification in Fram Strait region. Additionally, some modelling suggests that decreasing sea-ice cover may lead to increased import of Atlantic Waters—and thus, fixed N—to the Fram Strait region^98^. Therefore in addition to changes in the areal extent of ice-free ocean^27,99,100^ any future projection of primary production in the Fram Strait region is also hampered by poor knowledge of changes in nutrient supply as a result of the strong seasonal and inter-annual variability in current regimes^31,73^, changes in phytoplankton community composition and their nutrient requirements^57,65,101^. This compounds a poor mechanistic understanding of what drives elevated dFe and Si concentrations in the Transpolar Drift and how this will respond to future climate perturbations^19,45,75^. In addition, the relative importance of N_2_ fixation by diazotrophs in this region is poorly quantified^102–104^. Diazotrophs have particularly high Fe requirements, therefore the distribution of dFe, which in Fram Strait appears to be predominantly supplied by the EGC, could also be important for controlling their distribution and the rate of new fixed N introduced into this system by this process.

## Supplementary information

Supplementary information.

## Data Availability

All data used throughout this publication is accessible online. PS100 (GN05) physical oceanography data can be obtained from: https://doi.pangaea.de/10.1594/PANGAEA.871030 (ucCTD), and https://doi.pangaea.de/10.1594/PANGAEA.871028 (stainless steel CTD), and https://doi.pangaea.de/10.1594/PANGAEA.873158 (ship-based thermosalinograph). PS100 (GN05) macronutrient data can be obtained from: https://doi.pangaea.de/10.1594/PANGAEA.905347 (ucCTD), and https://doi.pangaea.de/10.1594/PANGAEA.879197 (stainless steel CTD). PS100 (GN05) bioassay data, and dissolved trace metal data will be made available on Pangaea database following this publication.
