# Supplementary material for: The influence of Arctic Fe and Atlantic fixed N on summertime primary production in Fram Strait, North Greenland Sea
Source: Sci Rep. 2020 Sep 17;10:15230. doi: 10.1038/s41598-020-72100-9 (PMC7499181; doi:10.1038/s41598-020-72100-9)
Supplement: Supplementary file 1 — Supplementary information. [file 41598_2020_72100_MOESM1_ESM.docx]

**Supplementary Information**

to

**The influence of Arctic Fe and Atlantic fixed N on summertime primary production in Fram Strait, North Greenland Sea**

Stephan Krisch^1^, Thomas J. Browning^1^, Martin Graeve^2^, Kai‑Uwe Ludwichowski^2^, Pablo Lodeiro^1^, Mark J. Hopwood^1^, Stéphane Roig^1^, Jaw-Chuen Yong^1,3^, Torsten Kanzow^2^, Eric P. Achterberg^1Ŧ^

^1^GEOMAR Helmholtz Centre for Ocean Research, Marine Biogeochemistry Division, 24148 Kiel, Germany.

^2^Alfred-Wegener-Institute for Polar and Marine Research, 27570 Bremerhaven, Germany.

^3^ Universiti Malaysia Terengganu, Faculty of Science and Marine Environment, 21030 Kuala Nerus, Terengganu, Malaysia.

*^Ŧ^ Correspondence and requests for materials should be addressed to*: eachterberg@geomar.de

**SI-1: Salinity-fixed N, Si and dFe relationships**


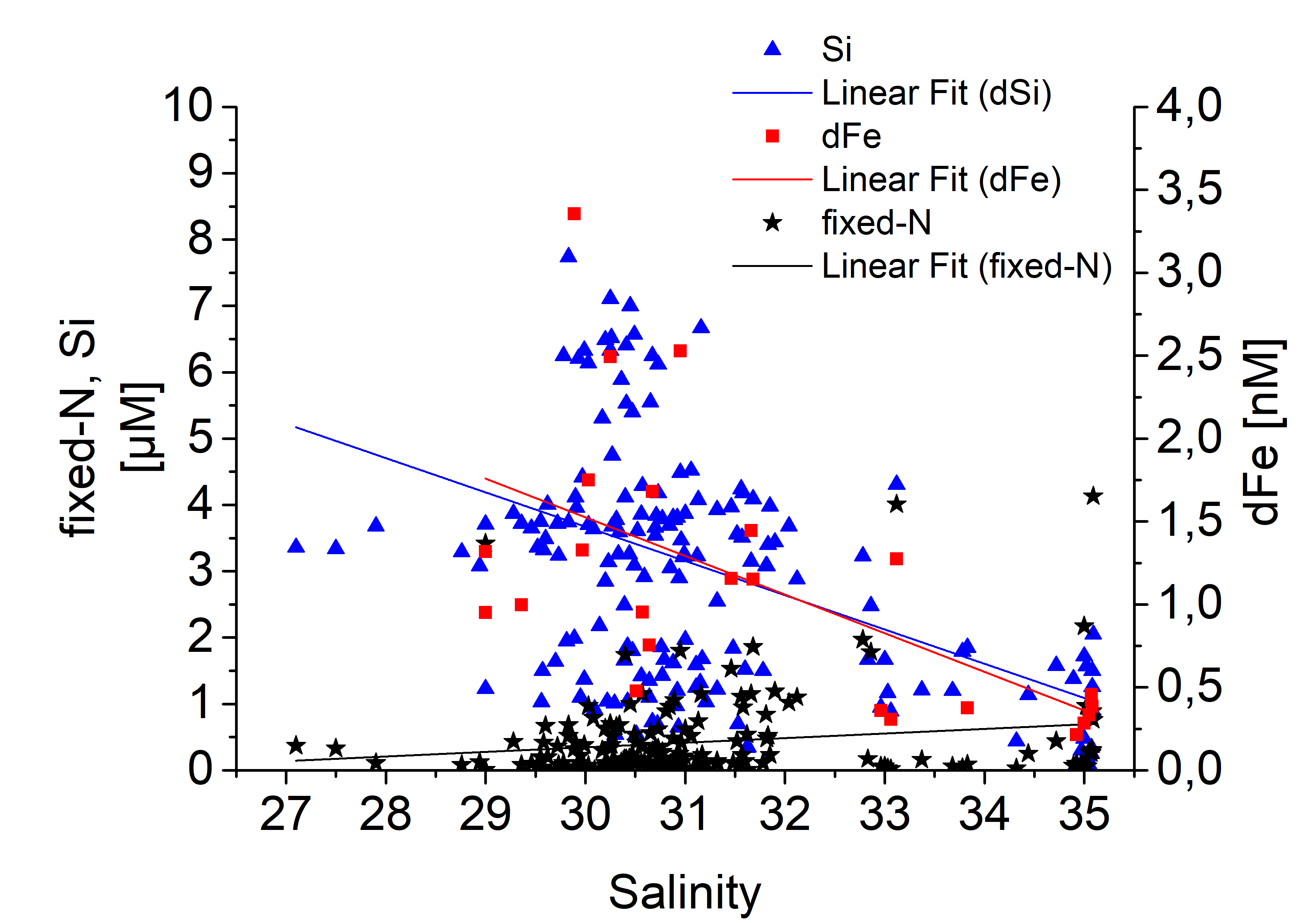


**Figure S1:** Fixed N, Si and dFe versus practical salinity for surface (10 m) waters in Fram Strait region. Standard error not shown for clarity but included in linear approximation through program-based direct weighing: [fixed N] = ‑1.73 (0.90) + 0.07 (0.03) S, [Si] = 19.2 (3.8) - 0.52 (0.12) S, and [dFe] = 8.52 (1.96) - 0.23 (0.06) S.

Figure plotted using OriginPro, version 9.1.0. (OriginLab Corporation, Northampton, MA, USA).

**SI-2: Profiles of potential temperature, fixed N and dFe**


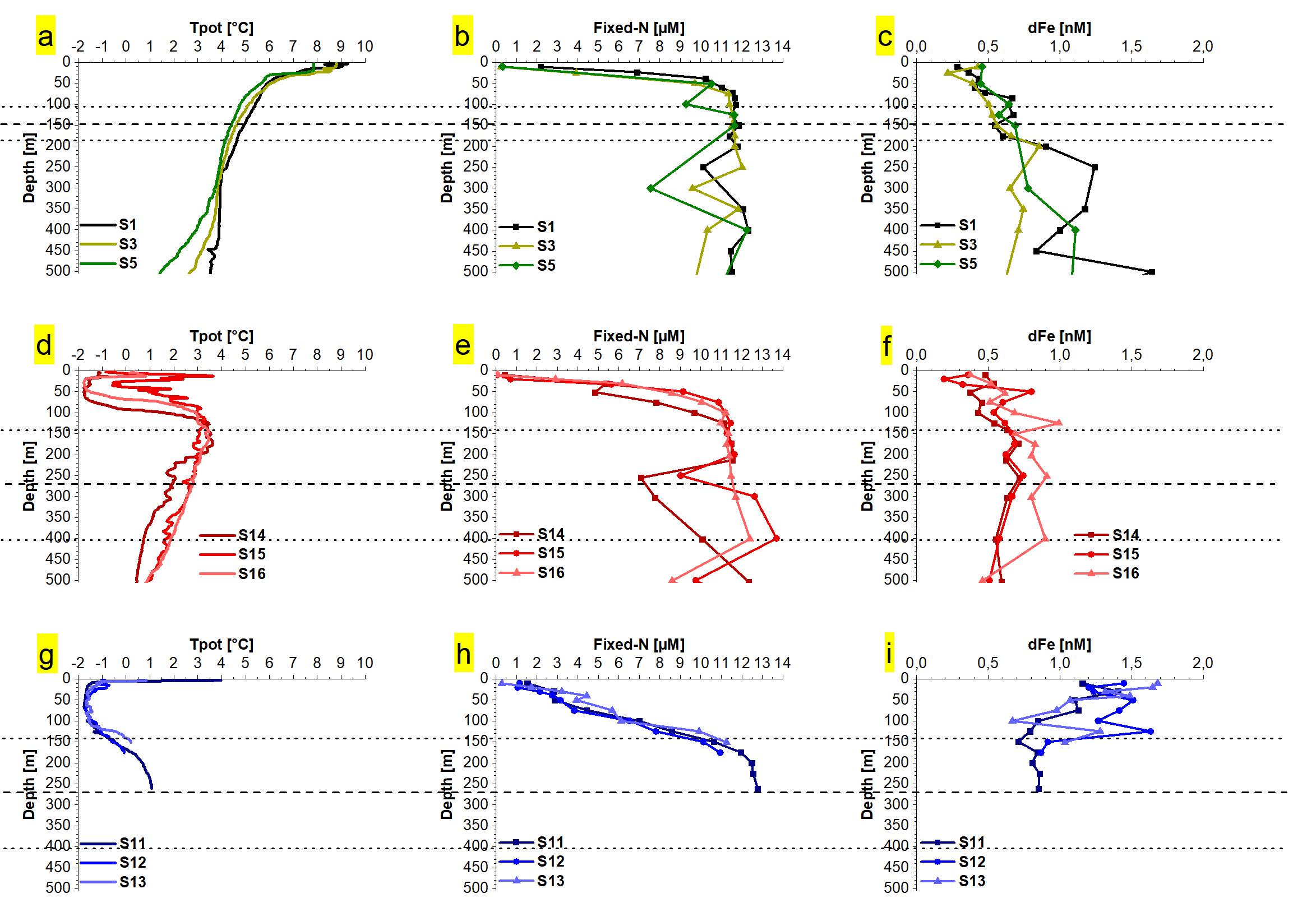


**Figure S2:** Vertical distribution (<500 m) of potential temperature (T_pot._), fixed nitrogen (fixed‑N) and dissolved Fe (dFe) of exemplary stations in (a‑c) the West Spitsbergen Current (top row, stations S1, S3 and S5), the East Greenland Current (d‑f) in western Fram Strait (middle row, S14‑16) and (g‑i) on the outer NE Greenland Shelf (bottom row, S11‑S13).

Dashed line indicates mean value (± standard deviation, dotted lines) of maximum climatological winter mixed layer depths (Dec-Mar) in the West Spitsbergen Current (a-c) and the East Greenland Current (d-i) obtained from de Boyer Montégut *et al.* (2004) between 76‑80°N, 20°W‑15°E. Definition of West Spitsbergen Current (surface salinity >34.8) and East Greenland Current (<34.8) according to surface salinity measurements (11m) from Polarstern cruises to Fram Strait region between 2004‑2019.

Figure plotted with OriginPro, version 9.1.0. (OriginLab Corporation, Northampton, MA, USA).
